# Vaccination with BNT162b2 and ChAdOx1 nCoV-19 Induces Cross-Reactive Anti-RBD IgG against SARS-CoV-2 Variants including Omicron

**DOI:** 10.3390/v14061181

**Published:** 2022-05-28

**Authors:** Daniela Gerges, Sebastian Kapps, Esperanza Hernández-Carralero, Raimundo Freire, Monika Aiad, Sophie Schmidt, Wolfgang Winnicki, Thomas Reiter, Sahra Pajenda, Alice Schmidt, Gere Sunder-Plassmann, Ludwig Wagner

**Affiliations:** 1 Division of Nephrology and Dialysis, Department of Medicine III, Medical University of Vienna, 1090 Vienna, Austria; daniela.gerges@meduniwien.ac.at (D.G.); sebastian.kapps@meduniwien.ac.at (S.K.); monika.aiad@students.boku.ac.at (M.A.); sophie.schmidt@akhwien.at (S.S.); wolfgang.winnicki@meduniwien.ac.at (W.W.); thomas.reiter@meduniwien.ac.at (T.R.); sahra.pajenda@meduniwien.ac.at (S.P.); alice.schmidt@meduniwien.ac.at (A.S.); gere.sunder-plassmann@meduniwien.ac.at (G.S.-P.); 2 Unidad de Investigacion, Hospital Universitario de Canarias-FIISC, 38320 La Laguna, Spain; esperanza.carralero@gmail.com (E.H.-C.); rfreire@ull.edu.es (R.F.); 3 Instituto de Tecnologías Biomedicas, Universidad de La Laguna, 38200 La Laguna, Spain; 4 Universidad Fernando Pessoa Canarias, 35450 Las Palmas de Gran Canaria, Spain

**Keywords:** Omicron, receptor-binding domain (RBD), cross-reactive antibodies, COVID-19, SARS-CoV-2, variants of concern

## Abstract

SARS-CoV-2 variants of concern (VOCs) have caused a significant increase in infections worldwide. Despite high vaccination rates in industrialized countries, the fourth VOC, Omicron, has outpaced the Delta variant and is causing breakthrough infections in individuals with two booster vaccinations. While the magnitude of morbidity and lethality is lower in Omicron, the infection rate and global spread are rapid. Using a specific IgG multipanel-ELISA with the spike protein’s receptor-binding domain (RBD) from recombinant Alpha, Gamma, Delta, and Omicron variants, sera from health-care workers from the Medical University of Vienna were tested pre-pandemic and post-vaccination (BNT162b2; ChAdOx1 nCoV-19). The cohort was continuously monitored by SARS-CoV-2 testing and commercial nucleocapsid IgG ELISA. RBD IgG ELISA showed significantly lower reactivity against the Omicron-RBD compared to the Alpha variant in all individuals (***p* < 0.001**). IgG levels were independent of sex, but were significantly higher in BNT162b2 recipients <45 years of age for Alpha, Gamma, and Delta (***p* < 0.001; *p* = 0.040; *p* = 0.004**, respectively). Pre-pandemic cross-reactive anti-Omicron IgG was detected in 31 individuals and was increased 8.78-fold after vaccination, regardless of vaccine type. The low anti-RBD Omicron IgG level could explain the breakthrough infections and their presence could also contribute to a milder COVID-19 course by cross-reactivity and broadening the adaptive immunity.

## 1. Introduction

As the world has been kept in suspense by the existing SARS-CoV-2 pandemic since the beginning of 2020, the developments of SARS-CoV-2 vaccines have been considered a great hope and have saved many lives so far. Nevertheless, despite high vaccination-coverage rates in industrialized countries, the SARS-CoV-2 variant Omicron causes a high disease incidence with breakthrough infections, which, however, appear to be milder [1]. It is unclear, to date, whether this is due to the cross-reactivity of immunized and convalescent individuals or to an overall lower virulence of the viral variant. The aim of this study was to compare cross-recognition of the spike protein receptor-binding domain (RBD)-specific IgG, which we previously detected as one of the most immunogenic regions of the virus [2] between the SARS-CoV2 variants Alpha, Gamma, Delta, and Omicron induced by vaccination or mild COVID-19 disease. In addition, these results were compared with pre-pandemic serologies from the same study participants. 

To date, several hypotheses have been proposed to explain Omicron-induced breakthrough infections in vaccinated individuals and in the presence of antibodies. It has been shown that the inactivated virus vaccine BBIBP-CorV booster immunization leads to neutralizing antibodies against Omicron variants [3], yet it appears that Omicron can evade pre-existing antibodies due to mutations in the RBD of the spike protein, the main target of neutralizing antibodies [4,5,6,7,8,9]. In Omicron, the binding motif of the RBD and the furin cleavage site are affected by mutations that influence binding and fusogenic potential [10,11].

Mutations in the RBD result in a higher affinity for ACE2, giving Omicron a higher probability of infecting a cell. However, Omicron has been reported to shift cellular tropism away from TMPRSS2-expressing cells, found primarily in the lower bronchial system, which would explain the primary upper respiratory tract symptoms of Omicron [9,12,13,14]. This results in lower morbidity and need for hospitalization in patients infected with Omicron compared to previous SARS-CoV-2 variants [1,15]. Its propensity to infect cells in the upper respiratory tract, coupled with its high affinity for ACE2, gives the virus an advantage in spreading more rapidly and easily than other variants.

However, it is not yet fully understood whether the high reinfection rates with the Omicron variant are due to waning immunity or to its mutation rate, which helps to circumvent immunity [7,16]. A recent study suggests that reinfection with Omicron mirrors the situation of seasonal coronavirus HCoV-229E, in which repeated infection of the same person with that virus over a lifetime is due to viral changes [17].

The aim of this study was to focus on the antibody recognition pattern within the RBD of the spike protein of the Alpha, Gamma, Delta, and Omicron variants of SARS-CoV-2 by examining IgG in a cohort of health-care workers in a nephrology department in Vienna, Central Europe, at a time point when Omicron was not yet detected in the region, in March 2020. The participants were exposed to patients who were immunosuppressed due to renal transplantation. Recently, it has been documented that vaccine-induced SARS-CoV-2 immunity in this patient group is inefficient at both the antibody level and in the T-cell response [18]. This makes the nephrology health-care workers a high-risk group to come into contact with infected individuals. Therefore, in this study, we focused on cross-reactive anti-Omicron IgG in the sera of individuals before the pandemic and after vaccination (BNT162b2 and ChAdOx1 nCoV-19). We also compared IgG reactivity against the Omicron RBD with the Alpha, Gamma, and Delta variants in individuals after vaccination. In addition, the sex and age dependence of IgG levels induced by vaccination was investigated. To our knowledge, this is the first study comparing anti-RBD Omicron IgG from the pre-pandemic period with post-vaccine conditions in a cohort of healthcare workers.

## 2. Materials and Methods

### 2.1. Sample Collection and Cryopreservation

**Figure 1:**
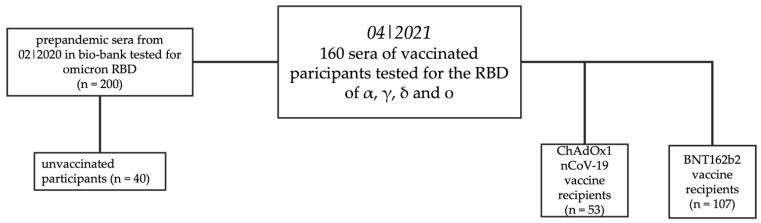
Flow chart of analyzed sera and vaccination status.

In March 2020, staff from the Department of Nephrology and Dialysis at the Medical University of Vienna were enrolled in a serological follow-up study. In total, 240 participants were enrolled in the study, signed an informed consent form, and allowed the publication of potentially interesting results. The study was reviewed by the ethics committee of the Medical University of Vienna, under the number 1357/2020, as part of the CONEC study (ClinicalTrials.gov no. NCT04347694). The first results were published in 2021 [19,20]. As the pandemic progressed through the different variants, 160 of the 240 individuals participated in vaccination as soon as BNT162b2 or ChAdOx1 nCoV-19 vaccines were available, which were administered in late January through March 2021 (Figure 1). Blood collection occurred within a two-week period starting on 14 March 2020, and later, post-vaccination sera collection started on 7 April 2021. Serum was separated by centrifugation and frozen in aliquots at −80 °C. For each participant, the date and type of vaccination and health-related information were recorded as indicated in the demographic table. A separate aliquot of serum was thawed from each participant who had been vaccinated to perform multipanel ELISA testing for RBD variants (Figure 1).

### 2.2. Protein Production

The DNA encoding for the receptor binding domains (RBD, 300–685 aa) were cloned into the pet-30a plasmid from the Alpha, Gamma, Delta, and Omicron variants of SARS-CoV-2 and sequenced by the Sanger method. Purified plasmids were transfected into BL21 DE3 *Escherichia coli* (*E. coli*). Outgrown clones were amplified in LB/Kan overnight at 37 °C. The next morning, LB/Kan culture was increased fivefold with Terrific Broth (Gibco A13743-01) and protein expression was induced with 200 µM IPTG and kanamycin. Protein expression was performed at 22 °C under constant shaking. After 24 h, *E. coli* cells were pelleted in a FIBERLite^®^ rotor (ThermoScientific, Waltham, MA, USA, F15-8 × 50 cy) and lysed in 7 M urea 0.1 M NaH_2_PO_4_, 0.01 M Tris Cl (pH 8). The lysate was sonicated for 10 s and incubated with the addition of benzonase endonuclease for 10 min at room temperature (RT). The insoluble material was pelleted at 12,000 g for 10 min at 4 °C. The pre-cleared lysate was passed over an Ni-NTA binding column (Qiagen, Hilden, Germany) by centrifugation at 2000× *g*. The loaded column was washed with 8 M urea, 0.1 M NaH_2_PO_4_, 0.01 M Tris-Cl (pH 8.0), 20 mM imidazole, followed by 8 M urea, 0.1 M NaH_2_PO_4_, 0.01 M Tris-Cl (pH 6.3). Finally, protein elution was performed with 200 µL 8 M urea, 0.1 M NaH_2_PO_4_, 0.01 M Tris-Cl (pH 4.5) containing 250 mM imidazole in three separate fractions. Each of them was analyzed by SDS-PAGE gels under reducing conditions and by spectrophotometry using a Nanodrop instrument.

### 2.3. ELISA

#### 2.3.1. RBD Alpha, Gamma, Delta, and Omicron Variant ELISA

ELISA plates (Nunc #464718) with 384 wells were coated with 8 ng of RBD variants Alpha, Gamma, Delta, or Omicron protein per well. The coating was performed overnight at 4 °C. The wells were blocked with blocking buffer (2× blocking reagent in PBS, 11500694001, Roche) for 30 min at RT on a rotating platform. After a brief wash with PBS in an automated ELISA washer (ELX50 Auto Strip Washer, Bio-Tek Instruments, Inc., Winooski, VT, USA), diluted sera were added in duplicate to each well to coat the Alpha, Gamma, Delta, and Omicron wells for each individual donor. After incubation on a shaking platform for one hour, the plates were washed with 150 µL TPBS three times. To detect the binding antibody, goat anti-human IgG (H + L) F(ab’)2 HRP (1:40,000) was diluted in assay buffer with goat serum (5% *V/V*) and incubated for 60 min at RT on a shaking platform. After another wash with TPBS, the chromogen/substrate mixture TMB was applied and reacted for 12 min in the dark. Subsequently, 50 µL of 2N-sulfuric acid was added and the ELISA was read at 450 nm using an ELISA reader (Synergy H1 Hybrid Reader, Bio-Tek, Inc.)

#### 2.3.2. Nucleocapsid ELISA

The test kit was purchased from ImmunoDiagnostics (IMD) Hong Kong, Hong Kong Special Administrative Region of the People’s Republic of China. The pre-coated ELISA plates were brought to RT before opening the sealed package. A positive sample included within the test kit was placed in duplicate in each ELISA plate. Participant sample sera were diluted 1:100 with assay buffer after thawing immediately before use. The plate was then incubated for 60 min at RT on a plate shaker. After three washing steps with 300 µL wash buffer in an automated ELISA washer, the IgG detection antibody pre-diluted in assay buffer was applied to the plate and incubated for 60 min at RT on a shaker platform. The ELISA was then developed using the TMB substrate/chromogen mixture provided with the test kit after the plate was re-washed as described above. Colour development performed in the dark was stopped by applying stop solution to each well. The ELISA was then read at 450 nm using an ELISA reader.

### 2.4. Statistical Analysis

Appropriate statistical tests were performed for each experiment, and statistical significance calculated using Prism (Prism 9 for macOS, Version 9.3.1 (350), Dennis Radushev, Macintosh Version by Software MacKiev 1994–2021 GraphPad Software, LLC for macOS, San Diego, CA, USA). To determine statistical significance without assuming Gaussian distribution, Kruskal–Wallis, followed by Dunn’s multiple comparisons test, were performed. Whereas, to evaluate differences between vaccines, two-way analysis of variance (ANOVA), followed by Sidak’s multiple comparisons test, were performed. Correlation coefficients and significances were obtained using the Spearman correlation method. A *p*-value < 0.05 was considered statistically significant. Statistical significance was noted as follows: * *p* < 0.05, ** *p* < 0.01, *** *p* < 0.001, **** *p* < 0.0001. Unless stated otherwise, the mean ± standard deviation (SD) for normally distributed samples are depicted. The median and the lower and upper quartiles are given for skewed data.

## 3. Results

### 3.1. Establishment of an Anti-IgG Multipanel ELISA against RBD Variants Alpha, Gamma, Delta, and Omicron

**Figure 2:**
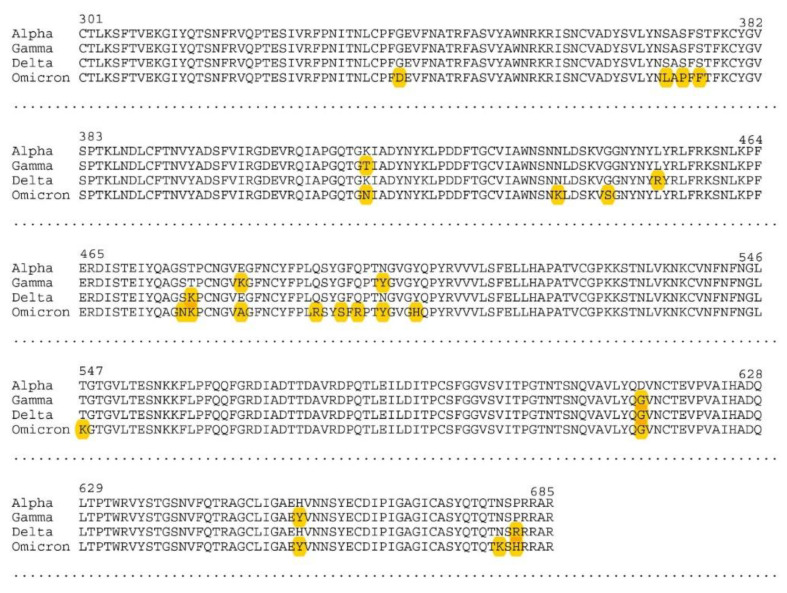
Alignment of the RBD part of the spike protein of SARS-CoV-2, with amino acid changes indicated in yellow when comparing variants Gamma, Delta, and Omicron with Alpha.

The RBD (300–685 aa) of the spike proteins of the SARS-CoV-2 variants was cloned into the pet-30a vector, and the protein was purified via its His-tag. The amino acid changes in the Gamma, Delta, and Omicron variants compared with Alpha in this RBD region are shown in Figure 2. Equal amounts of the recombinant protein from each variant were applied to 384-well ELISA plates in separate rows so that each participant serum could be tested on the same assay plate for all four variants. This ensured absolute intra-variant comparability for each subject.

IgG detection of RBD Alpha was compared with that of Gamma, Delta, and Omicron in the vaccinated cohort (*n* = 160). Out of these, 107 participants had received two BNT162b2 (Pfizer) doses and 53 had received the first dose of ChAdOx1 nCoV-19 (Oxford/AstraZeneca), with all of them at least 14 days prior to the blood sampling. Quantitative data are given as mean ± standard deviation; qualitative data are provided as counts (%).

**Figure 3:**
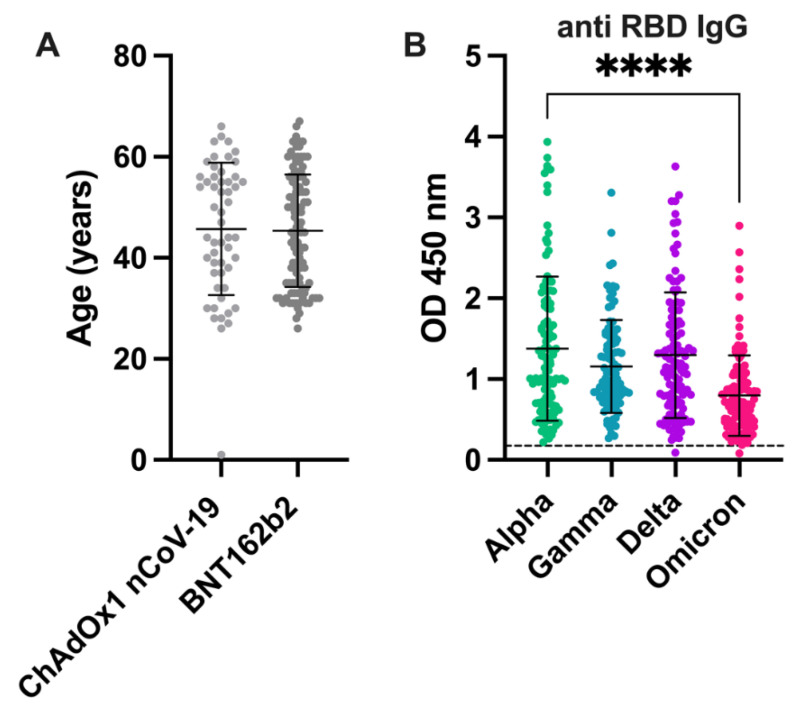
Age distribution of vaccinees and anti-RBD IgG levels against SARS-CoV-2 variants. (**A**) Age distribution of participants receiving ChAdOx1 nCoV-19 (Oxford/AstraZeneca) or BNT162b2 (Pfizer); (**B**) Anti-RBD-domain IgG levels detected against the variants Alpha, Gamma, Delta, and Omicron after vaccination regardless of vaccine type; **** *p* < 0.0001, hyphenated line represents the test cut-off.

In clinical studies, the severity of the disease was associated with older age [21] due to various factors [22]. To clarify whether there was a difference between the age of the participants when vaccinated with BNT162b2 or ChAdOx1 nCoV-19 and whether this could affect the adaptive immune response, we analyzed the age distribution among the participants. As indicated in Figure 3A and Table 1, there was no difference in the average age of the participants for either group (BNT162b2: 45.7 ± 11.9 years vs. ChAdOx1 nCoV-19: 45.7 ± 13.1 years; *p* = 0.984).

There were no differences in the IgG reactivity of Gamma and Delta compared to Alpha, although significantly reduced IgG reactivity was detected against Omicron when compared to Alpha (*p* < 0.0001) (Figure 3B).

**Figure 4:**
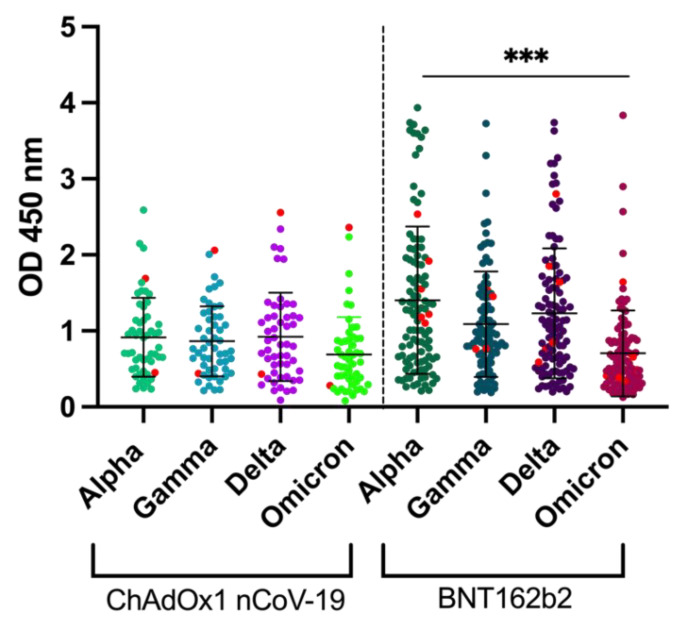
Intervariant differences of RBD-specific IgG of ChAdOx1 nCoV-19 (Oxford/AstraZeneca) recipients compared with BNT162b2 (Pfizer). Individuals who tested positive in the nucleocapsid ELISA and were classified as convalescent are marked in bright red. (*** *p* < 0.001).

When the vaccinated individuals were compared, ChAdOx1 nCoV-19 recipients were found to have significantly lower IgG levels against the RBD across all four variants compared to BNT162b2. This must be explained by the fact that all ChAdOx1 nCoV-19-recipients received the test blood collection 14 days after the first vaccination while the obligatory second vaccination was still pending (Figure 4). Among the vaccine recipients were 31 individuals who had undergone COVID-19 after pre-pandemic sampling as their sera contained IgG that recognized the nucleocapsid protein, not that present in the vaccine, but in the SARS-CoV-2 infection.

**Figure 5:**
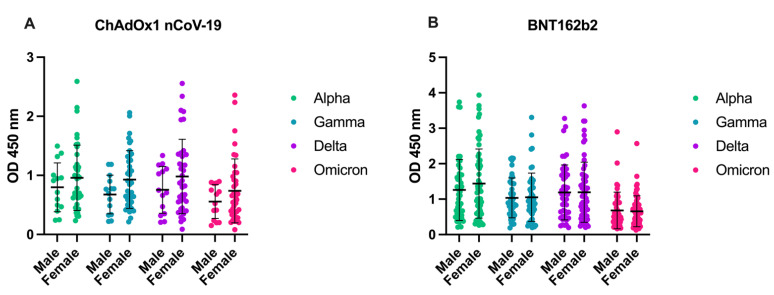
Gender dependence of RBD variant–specific IgG induction following vaccination with either ChAdOx1 nCoV-19 (Panel **A**) or BNT162b2 (Panel **B**).

As it is known from clinical data that gender differences in terms of morbidity and mortality among COVID-19 patients exist, the gender effect on IgG production was evaluated and no differences were found (*p* = 0.779; *p* = 0.388; *p* = 0.503; *p* = 0.700) for Alpha, Gamma, Delta, and Omicron, respectively (Figure 5).

**Figure 6:**
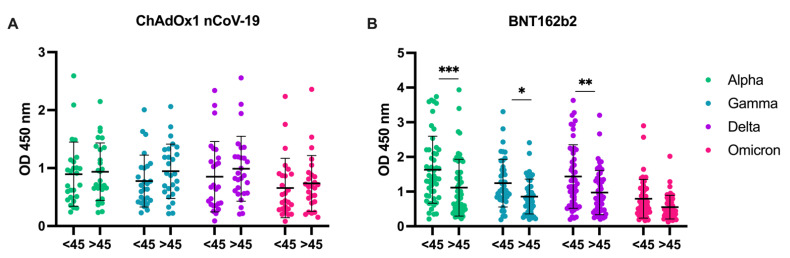
Age dependence of anti-RBD IgG against variants Alpha, Gamma, Delta, and Omicron. (**A**) Participants who received the first dose of ChAdOx1 nCoV-19. (**B**) Participants who received the booster dose of BNT162b2; * *p* < 0.05; ** *p* < 0.01; *** p < 0.001.

As it has been observed that older individuals are more likely to experience severe COVID-19, the age dependence of IgG antibody production and its cross-reactivity within the variants was evaluated for BNT162b2 and ChAdOx1 nCoV-19 vaccine recipients (Figure 6A,B). The age cut-off was chosen at 45 years as this presented the mean age of the participants (Figure 3A, Table 1). Participants treated with the BNT162b2 vaccine who were younger than 45 years of age developed significantly higher IgG reactivity against the Alpha, Gamma, and Delta variants compared to the group older than 45 years (*p* < 0.001; *p* = 0.040; *p* = 0.004, respectively). There was no age-dependent statistically significant difference in the IgG reactivity against Omicron in BNT162b2 vaccinees or against all variants in ChAdOx1 nCoV-19 vaccinees (Figure 6B).

### 3.2. RBD Variant IgG Levels Affected by Moderate COVID-19

Of the 160 participants, 31 tested positive with the anti-nucleocapsid IgG ELISA test, which was consistent with clinical data in the records at the time of blood collection, and all 31 participants had experienced mild-to-moderate COVID-19 disease. However, no significant association was observed between IgG reactivity against RBD variants Alpha, Gamma, Delta, and Omicron compared to anti-nucleocapsid IgG reactivity in the IMD ELISA test.

### 3.3. Evaluating RBD IgG Cross-Reactivity in Pre-Pandemic Sera and the Increase in IgG Reactivity against Omicron Variant after BNT162b2 or ChAdOx1 nCoV-19 Vaccination

**Figure 7:**
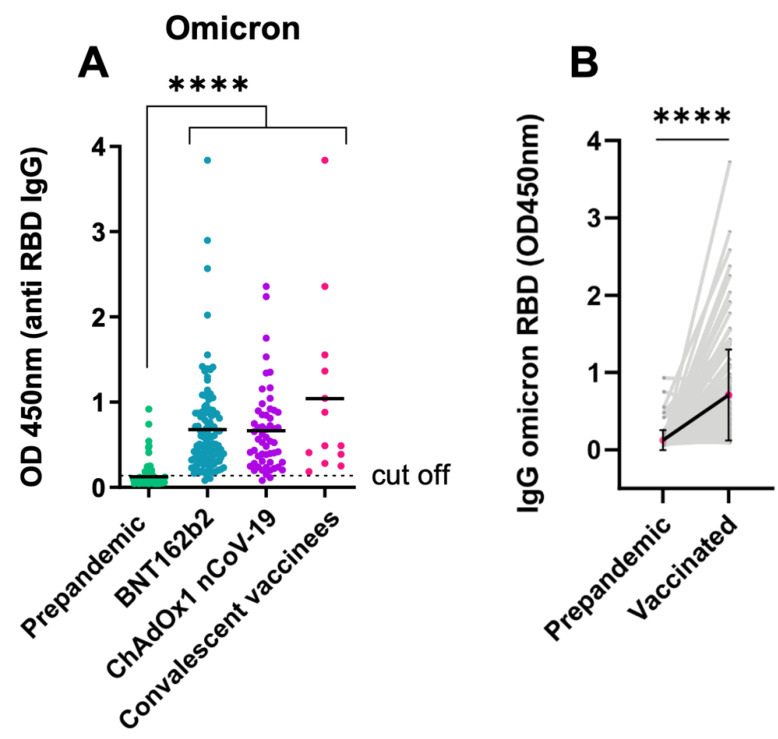
Cross-reactive IgG antibody levels could be found in several pre-pandemic sera but increased significantly after vaccination. (**A**) Anti-Omicron IgG increasing from the pre-pandemic status upon vaccination with BNT162b2 (Pfizer) or ChAdOx1 nCoV-19 (Oxford/AstraZeneca) vaccine and/or having undergone mild-to-moderate COVID-19 in healthcare workers. (**B**) Anti-Omicron-RBD IgG increase from pre-pandemic levels, exhibiting cross-reactivity and increase following vaccination with the BNT162b2 or ChAdOx1 nCoV-19 vaccines. For paired samples, Wilcoxon test was applied; **** *p* < 0.0001.

In a retrospective analysis of more than 16,000 COVID-19 patients, a cohort with a documented history of endemic coronavirus infection was found to have lower disease progression and lethality than a cohort without endemic coronavirus infection [23]. In addition, 10 out of 50 pregnant women sampled in 2018 showed SARS-CoV-2-reactive IgG [24]. This prompted us to analyse 200 individual sera stored in the biobank from a time point before the pandemic. Among the vaccine samples, 128 individuals had a serum sample in the biobank from March 2020, when Vienna was still at the early stages of the first COVID-19 wave. These individuals had not been infected with SARS-CoV-2 up to that point because they had not been in contact with COVID-19 patients or infected individuals elsewhere, according to their medical histories, and also did not test positive for nucleocapsid. Surprisingly, 30 of them showed IgG reactivity against Omicron (Figure 7A). Only those participants who showed an anti-Omicron IgG OD value 2-fold greater than the test threshold at the titer of 1:100 were considered positive. This level increased eightfold (8.78) following vaccination for Omicron (Figure 7B).

In the subgroup analysis where pre-pandemic values were available for vaccinees, fully vaccinated BNT162b2 recipients showed a 7.71-fold (*n* = 76) increase, whereas half vaccinated ChAdOx1 nCoV-19 recipients showed a 6.47-fold (*n* = 22) increase in anti-Omicron RBD IgG. Anti-Omicron IgG in COVID-19 convalescents in combination with vaccine (*n* = 6) showed a 14.88-fold increase compared with baseline cross-reactive anti-Omicron IgG (Figure 7A). For 86 individuals in the same subgroup (pre-pandemic versus post-vaccination) the increases against Alpha, Gamma, and Delta variants were also determined following vaccination with BNT162b2 or ChAdOx1 nCoV-19 vaccines and revealed increases of 10.53-fold, 8.66-fold, and 6.11-fold, respectively (Supplementary Figure S1).

## 4. Discussion

In this study, IgG antibodies against the RBD domain of the spike protein were investigated after vaccination in a cohort of health-care workers at a nephrology department in Vienna. Cross-reactivity between the Alpha, Gamma, Delta, and Omicron variants was examined after full-vaccination with the BNT162b2 (Pfizer) and after half-vaccination with one dose of ChAdOx1 nCoV-19 (Oxford/AstraZeneca). Particular attention was paid to IgG reacting with the RBD of the Omicron variant in the ELISA setup. In this regard, low cross-reactivity was observed in participants in the pre-pandemic phase of the observation period induced by seasonal coronaviruses [25]. Vaccination-induced IgG levels against Omicron were detected in more than 95% of participants. The median RBD IgG levels of those who had experienced COVID-19 and were later vaccinated were only moderately higher than those vaccinated with BNT162b2. IgG response against all variants was not sex-dependent but decreased with age in BNT162b2 recipients older than 45 years.

There is still debate as to why vaccinated individuals have Omicron breakthrough infections and Omicron-infected individuals rarely develop severe COVID-19 requiring intensive care [1,5]. This study has yielded at least two remarkable results. First, vaccination with BNT162b2 or ChAdOx1 nCoV-19 induced lower IgG reactivity against Omicron compared to Alpha, Gamma, and Delta. Nevertheless, 97% of the participants showed positive values, indicating the presence of cross-reactive IgG antibodies up to an end-titer of 1:1000 (data not shown). The second interesting picture that emerged from the screening of 200 pre-pandemic sera for the RBD variants was the observation of cross-reactivity against Omicron due to endemic coronavirus infections.

These two observations could explain, from an adaptive immunity perspective, why individuals infected with Omicron develop less severe COVID-19, as the presence of cross-reactive antibodies from either endemic coronaviruses or SARS-CoV-2 vaccines could reduce the number of cells infected with the virus. In addition, the presence of such memory B cells could rapidly mature to form plasma cells and elicit the antiviral-specific T-cell response [26,27].

There is ongoing discussion whether cross-reactive endemic coronavirus antibodies could cause disease amplification in the setting of a SARS-CoV-2 infection [28]. Our data on cross-reactive RBD IgG antibodies in all the participants having undergone COVID-19 tend to argue against such an effect. In this context, it is interesting to note that non-human primates developed cross-reactive spike IgG antibodies to seasonal coronaviruses when vaccinated with a SARS-CoV-2 spike-encoding DNA vaccine and later being challenged with SARS-CoV-2 [29]. In humans, seroconversion to human seasonal coronavirus occurs in childhood [30] and it is refreshed by repetitive infections throughout life, most likely due to waning immunity and changes in the viruses as they recirculate annually [17]. SARS-CoV-2-neutralizing antibodies have been detected in SARS-CoV-2 uninfected individuals with the spike-specific binding site having been located at the S2 region by a soluble S2 competition assay [24] and epitope-resolved profiling [25].

Remarkable immune protection against the porcine epidemic diarrhea virus (PEDV), a coronavirus, was demonstrated in piglets by colostrum. The dams from which the milk originated had been fed corn containing spike protein during their gestation. Milk-transmitted antibodies reduced disease when piglets were exposed to this coronavirus [31]. Determining whether an oral or mucosal vaccine is effective against SARS-CoV-2 by using various immunogenic SARS-CoV-2 variant proteins is still in the preclinical stage [32].

In this study, the anti-nucleocapsid IgG levels behaved differently when compared to the anti-RBD IgG levels of the SARS-CoV-2 variants. One of the possible explanations is that anti-nucleocapsid IgG tends to exhibit different kinetics than anti-spike IgG. However, the most likely reason is that the vaccine elicits an immune response that promotes the production of RBD-specific IgG, with the RBD being the vaccine immunogen. In contrast, the nucleocapsid is an early immunogen in infection that is no longer present in convalescent individuals, and nucleocapsid serology is dominant in the early phase of infection but declines similarly [19] to anti-spike IgG levels [16].

Testing for neutralizing anti-Omicron antibodies in this cohort would have been desirable but due to limited access to a biosafety level 3 facility was not possible in this research setting. This represents a limitation of this study. It is reasonable to assume, however, that the Omicron variant is not neutralized by the presence of a small amount of IgG, although individuals with high titers may have a neutralizing capacity [9].

In conclusion it is conceivable that the low level of Omicron-specific anti-RBD IgG both enables the possibility of breakthrough infections, but, on the other hand, the mere presence of these antibodies may contribute to the milder course of COVID-19 in affected individuals.

## Figures and Tables

**Table 1:** Demographic characteristics of the vaccinated group.

Participants (n)	ChAdOx1 nCoV-19 (*n* = 53)	BNT162b2 (*n* = 107)	All(*n* = 160)	*p*-Value
**Age (years)**	45.7 ± 11.9	45.7 ± 13.1	45.3 ± 11.1	0.984
**Female sex—n (%)**	39 (73.6)	60 (56.1)	99 (61.9)	**0.038**

Bold in *p*-values indicates significance.

## Data Availability

The authors confirm that the data supporting the findings of this study are available within the article and/or its Supplementary Materials.

## Supplementary Materials

The following supporting information can be downloaded at: https://www.mdpi.com/article/10.3390/v14061181/s1, Figure S1: RBD region–specific IgG increase from pre-pandemic to postvaccine against Alpha, Gamma, and Delta variant.
