# Supplementary material for: Vaccination with BNT162b2 and ChAdOx1 nCoV-19 Induces Cross-Reactive Anti-RBD IgG against SARS-CoV-2 Variants including Omicron
Source: Viruses. 2022 May 28;14(6):1181. doi: 10.3390/v14061181 (PMC9231407; doi:10.3390/v14061181)
Supplement: Supplementary file 1 [file viruses-14-01181-s001.zip › viruses-1728081-supplementary.pdf]

Supplementary to Manuscript

Vaccination with BNT162b2 and ChAdOx1 nCoV-19 induce cross-reactive anti-RBD IgG against SARS-CoV2 variants including Omicron

Daniela Gerges[1], Sebastian Kapps[1], Esperanza Hernández[2,3,4], Raimundo Freire[2,3,4], Monika Aiad[1], Sophie Schmidt[1], Wolfgang Winnicki[1], Thomas Reiter[1], Sahra Pajenda[1], Alice Schmidt[1], Gere Sunder-Plassmann[1], and Ludwig Wagner[1]*

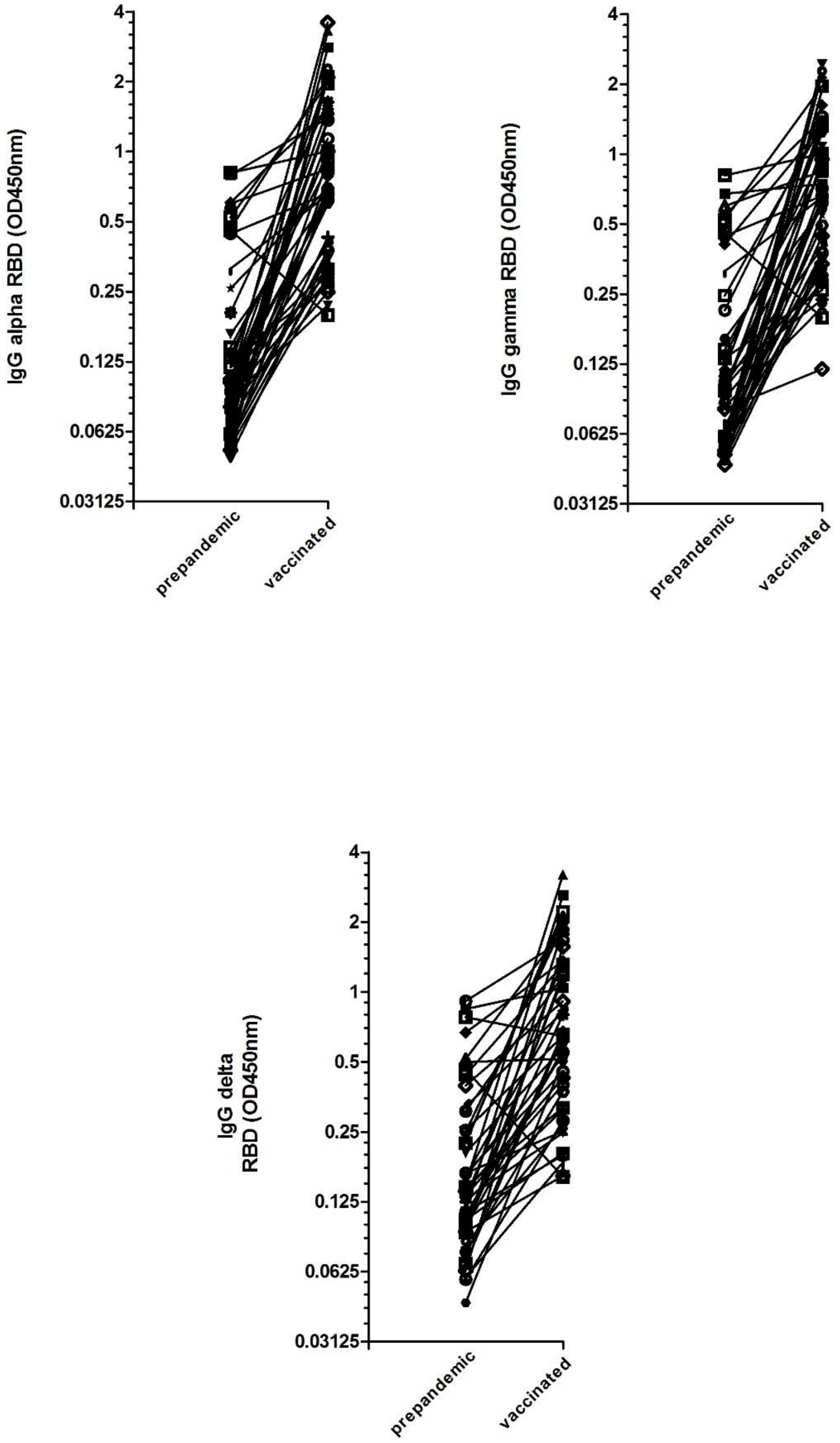

Supplementary Figure S1. RBD IgG cross-reactivity at the pre-pandemic testing and increase of IgG reactivity against Alpha, Gamma, and Delta RBD following vaccination with the BNT162b2 or ChAdOx1 nCoV-19 vaccines.
